# Single-Chain Polymer Nanoparticles Targeting the Ookinete Stage of Malaria Parasites

**DOI:** 10.1021/acsinfecdis.2c00336

**Published:** 2022-12-14

**Authors:** Naomi M. Hamelmann, Jan-Willem D. Paats, Yunuen Avalos-Padilla, Elena Lantero, Lefteris Spanos, Inga Siden-Kiamos, Xavier Fernàndez-Busquets, Jos M. J. Paulusse

**Affiliations:** † Department of Molecules and Materials, MESA+ Institute for Nanotechnology and TechMed Institute for Health and Biomedical Technologies, Faculty of Science and Technology, University of Twente, P.O. Box 217, 7500 AE Enschede, The Netherlands; ‡ The Barcelona Institute of Science and Technology, Institute for Bioengineering of Catalonia (IBEC), Baldiri Reixac 10−12, ES-08028 Barcelona, Spain; § Barcelona Institute for Global Health (ISGlobal, Hospital Clínic-Universitat de Barcelona), Rosselló 149-153, ES-08036 Barcelona, Spain; ∥ Institute of Molecular Biology and Biotechnology, FORTH, N. Plastira 100, 700 13 Heraklion, Greece; ⊥ Nanoscience and Nanotechnology Institute (IN2UB, Universitat de Barcelona), Martí i Franquès 1, ES-08028 Barcelona, Spain

**Keywords:** single chain polymer nanoparticles, Plasmodium berghei, drug-conjugate, atovaquone, intramolecular crosslinking, thiol-Michael addition

## Abstract

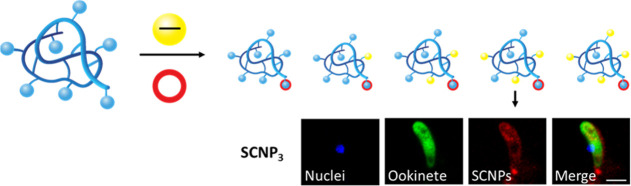

Malaria is an infectious disease transmitted by mosquitos, whose control is hampered by drug resistance evolution in the causing agent, protist parasites of the genus *Plasmodium*, as well as by the resistance of the mosquito to insecticides. New approaches to fight this disease are, therefore, needed. Research into targeted drug delivery is expanding as this strategy increases treatment efficacies. Alternatively, targeting the parasite in humans, here we use single-chain polymer nanoparticles (SCNPs) to target the parasite at the ookinete stage, which is one of the stages in the mosquito. This nanocarrier system provides uniquely sized and monodispersed particles of 5–20 nm, via thiol-Michael addition. The conjugation of succinic anhydride to the SCNP surface provides negative surface charges that have been shown to increase the targeting ability of SCNPs to *Plasmodium berghei* ookinetes. The biodistribution of SCNPs in mosquitos was studied, showing the presence of SCNPs in mosquito midguts. The presented results demonstrate the potential of anionic SCNPs for the targeting of malaria parasites in mosquitos and may lead to progress in the fight against malaria.

Malaria is one of the deadliest infectious diseases, caused by the protist parasite *Plasmodium* and transmitted by *Anopheles* mosquitos. Globally, there were an estimated 241 million malaria cases in 2020 in 85 countries, increasing from 227 million in 2019.^1^ The available arsenal of antimalarial drugs is insufficient to progress toward the eradication of the disease, a scenario that is worsened by the rampant evolution of resistance by the causing agent of malaria.^1^ The unmet medical and patient need of malaria eradication will not be achieved unless the targeted delivery of new drugs is vastly improved. To overcome these challenges multiple strategies are explored, including a combination of drug therapy and controlled drug delivery. Actually, the implementation of novel delivery approaches is less expensive than developing new antimalarial drugs and may even optimize the rate of release of current and novel compounds.^2^ In controlled drug delivery, the efficacy of therapeutics is optimized by utilizing nanoparticles (NPs). These transport the therapeutics efficiently to the target location in the body, also leading to a reduction of side effects. This approach is receiving growing interest for application against malaria, as it potentially uses a low dosage of therapeutics with increased accumulation at the target site, and minimizing the evolution of resistant parasite strains.^3^ Several NP systems have been investigated for their application to human malaria therapy, such as dendrimers,^4−6^ polymer micelles,^7^ liposomes,^8−13^ hydrogels,^14,15^ poly(amidoamine)-based NPs,^16,17^ zwitterionic self-assembled NPs,^18^ and glucose-conjugated gold NPs.^19^ A different antimalarial strategy involves transmission blocking vaccines (TBVs), which aim to inhibit the transmission of malaria from the vector to humans.^20−22^ TBVs aim to induce the development of antibodies in human blood that when taken up by mosquitoes will block the parasite in the mosquito. Especially in the early stages, comprising development to the ookinete stage (the motile zygote), constitute a bottleneck in the parasitic stages of the mosquito.^23,24^ However, this approach still requires the administration of vaccines to humans.

In recent years, research has been conducted on targeting the parasite at the ookinete stage by designing strategies to deliver nanocarriers directly to mosquitoes through meals in attractant reservoirs. This strategy would not require administration to humans, thus eliminating the need for the extensive clinical trials that usually preclude the development of antimalarial medicines because of the increase in development costs. In a recent work, we have shown targeting and transmission blockage of ookinetes by heparin.^25^ Following a membrane feeding assay of mosquitos using heparin, the oocyst counts were reduced by up to 37% as compared to the controls for non-modified heparin and reduced by 29% for hypersulfated heparin.

Polymer NPs provide an interesting opportunity to deliver anti-malarial agents to the ookinete. Control over surface modification is key to the functionalization of NPs for targeting and in this respect, single-chain polymer NPs (SCNPs) have shown promising potential for targeted drug delivery.^26−28^ SCNPs are intramolecularly cross-linked polymer chains and their properties are highly dependent on their precursor polymers.^29,30^ Narrow particle populations are achieved with diameters ranging from 5 to 20 nm when employing precursor polymers prepared via controlled/living polymerization techniques.^31^ In previous works, the synthesis of glycerol-SCNPs was demonstrated via thiol-Michael addition in either organic or aqueous solution to encapsulate a model drug.^32^ SCNPs formed via the organic route were rendered water-soluble and their biocompatibility was demonstrated. This strategy enables drug encapsulation largely irrespective of the lipo- or hydrophilicity of drug molecules. However, introduction of new functionalities on the SCNP surface by using functional monomers is a cumbersome approach, as incompatibilities with the cross-linking chemistry may occur. Post-formation functionalization of polymers^33,34^ and NPs^35^ is a straightforward and modular approach to incorporate functionality while maintaining properties such as a size constant. We recently reported on the highly specific surface modification of SCNPs through the use of pentafluorophenyl (PFP)-activated ester groups.^27^ The PFP-SCNPs were readily functionalized with a variety of small molecules and peptides, without markedly affecting the NP size.

Because heparin, which has the highest negative charge density of all known biomolecules,^36^ has been demonstrated to successfully target ookinetes, we hypothesized that negatively charged SCNPs are a promising candidate for targeting ookinetes. Here, we report the synthesis of glycerol-SCNPs and their post-formation functionalization with increasing amounts of succinic anhydride, resulting in increasing anionic surface charge. Additionally, a fluorescent label is conjugated onto the particles for in vitro and in vivo analyses. The biodistribution behavior of SCNPs is evaluated in mosquitos. Ookinete targeting efficiency is investigated as a function of SCNP surface charge by flow cytometry and confocal laser scanning microscopy (CLSM). An anti-malarial agent, atovaquone, is conjugated as a prodrug onto the glycerol-SCNPs and drug delivery is evaluated ex vivo on ookinete formation.

## Results and Discussion

**Figure 1:**
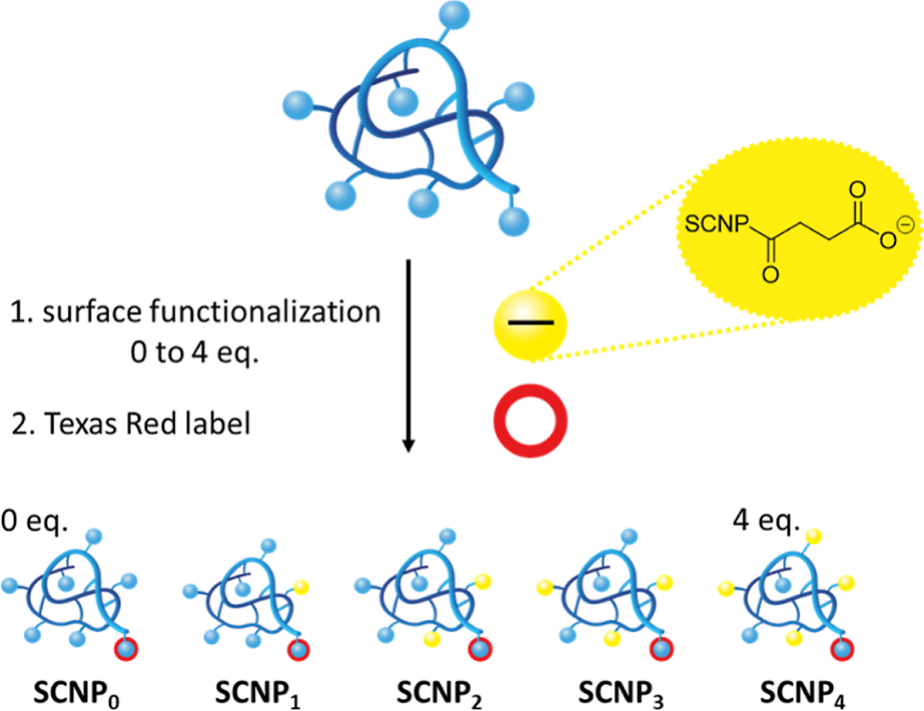
Schematic representation of SCNP surface functionalization with succinate groups and Texas Red labeling.

Glycerol-SCNPs were synthesized as described earlier^32^ via thiol-Michael addition through the slow addition of a thiol-functional co-polymer to a solution containing an acrylate-based cross-linker. Prior to particle formation, solketal moieties on the co-polymer were hydrolyzed to render the polymers water soluble. Subsequently, thiol functionalities were deprotected, followed by the cross-linking reaction utilizing poly(ethylene glycol) (PEG)-diacrylate as a (PEGDA) cross-linker (see Figure S1). Dynamic light scattering (DLS) measurements revealed a particle size of 8.3 nm (Figure S3) and STEM measurements confirmed an average size of ca. 10 nm (Figure S4). The surface of the glycerol-SCNPs was functionalized by the conjugation of increasing amounts of succinate groups onto the alcohol moieties to obtain a set of negatively charged particles (**SCNP**_**0 to 4**_, see Figure 1).

**Figure 2:**
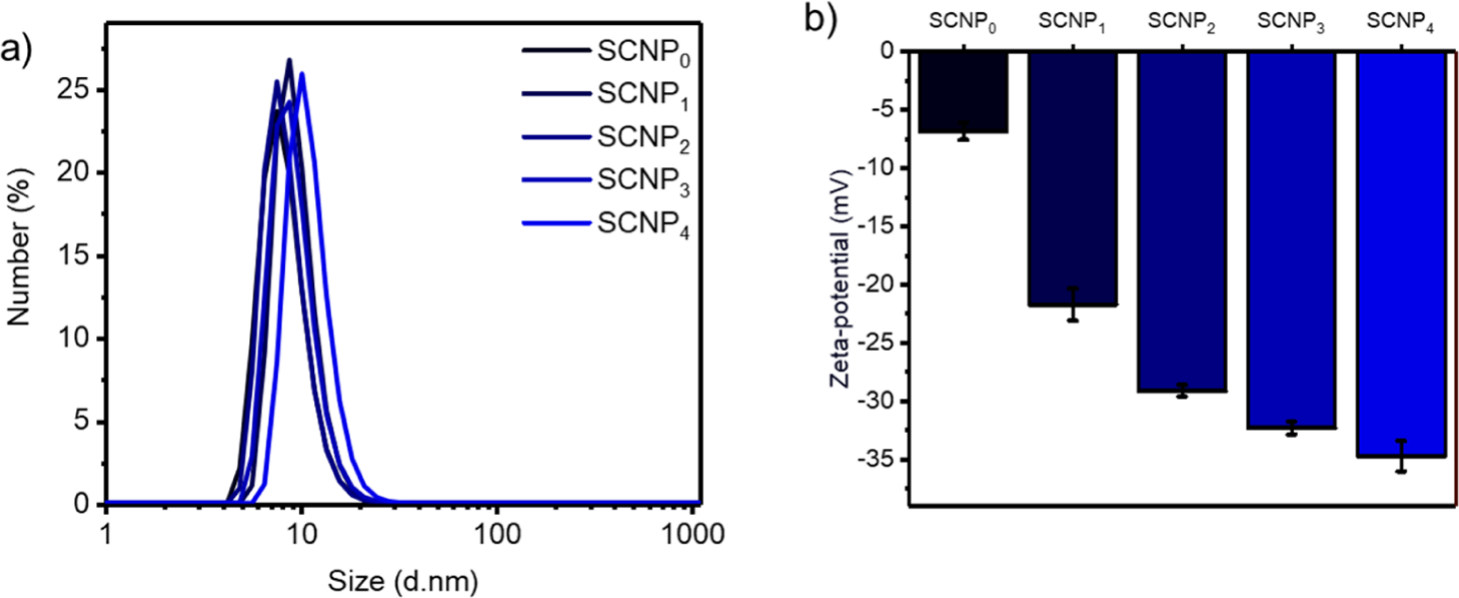
(a) Size of **SCNP**_**0-4**_ measured by DLS and (b) surface charge of **SCNP**_**0-4**_.

The conjugation of succinic anhydride was followed by ^1^H NMR spectroscopy, which showed an increasing signal at 2.2 ppm corresponding to the resulting succinate (Figure S5). In Figure 2, size according to DLS and zeta potential values of the SCNP set are shown. The SCNPs were monodisperse with a diameter of around 10 nm. The addition of increasing amounts of succinate groups did not noticeably affect the particle size. **SCNP**_**0**_ displayed a zeta potential of −6.8 ± 0.8 mV, while the introduction of succinate groups resulted in decreasing zeta potential values to as low as −34.7 ± 1.3 mV. For the evaluation of the targeting abilities of the SCNPs, a fluorescent Texas Red label or fluorescein label was conjugated onto the particles. The fluorescence signal of the Texas Red label measured by size exclusion chromatography (SEC) of **SCNP**_**0**_ co-elutes well with the refractive index (RI) signal, indicating successful conjugation of the Texas Red label (Figure S6).

**Figure 3:**
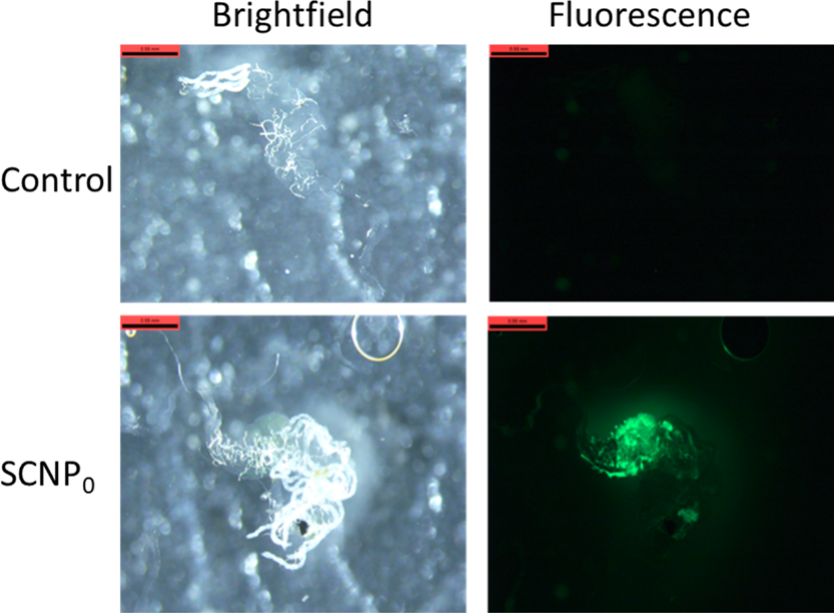
Dissected mosquito midguts imaged under a stereoscope after 3 days of feeding with fluorescein-labeled **SCNP**_**0**_. The SCNPs were provided in the sugar meal. The control was fed only sugar. Brightfield images are shown on the left, with the fluorescent signal of the **SCNP**_**0**_ shown on the right. The samples were imaged using the same settings. Scale bar 0.55 mm.

In order to target *Plasmodium* ookinetes, SCNPs are required to be taken up into the midguts of mosquitos. The biodistribution of nanocarriers can be studied by feeding mosquitos with sugar meals containing NPs. In previous works, PAAs were fed to mosquitos and their location was analyzed by fluorescence microscopy after dissecting the mosquitos.^17^ The biodistribution behavior of fluorescein-labeled **SCNP**_**0**_ was investigated by feeding the particles in 10% sucrose meals to mosquitos over the course of 3 days. Afterward, the midgut and salivary glands of the mosquitos were dissected. In Figure 3, the dissected midguts are shown. The fluorescent signal of **SCNP**_**0**_ was only detected in the midguts, demonstrating successful feeding to mosquitos and that SCNPs can reach the target site in the mosquito to enable further targeting of early mosquito stages including ookinetes.

**Figure 4:**
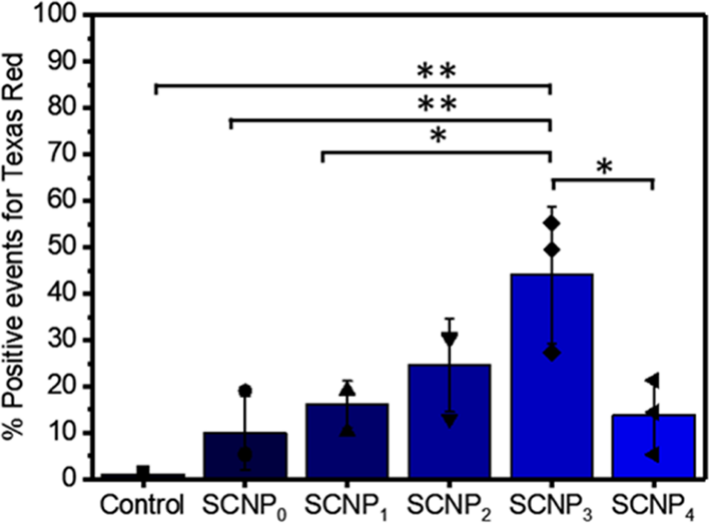
Percentage of positive binding events for SCNPs in the ookinete population measured by flow cytometry. *: *p* ≤ 0.05 and **: *p* ≤ 0.01.

The influence of anionic surface charge of SCNPs on the targeting of ookinetes was evaluated using in vitro cultured ookinetes from mouse blood infected with the *Plasmodium berghei* circumsporozoite protein and TRAP (thrombospondin-related adhesive protein)-related protein (CTRP)-green fluorescent protein (GFP) transgenic line,^37^ which expresses GFP at the ookinete stage. Ookinete targeting of the Texas Red-labeled SCNPs was quantified by flow cytometry after 1 h of incubation. In Figure 4, a stepwise increase in ookinete binding was observed for **SCNP**_**0**_ to **SCNP**_**3**_. Interestingly, the ookinete binding of **SCNP**_**4**_ is lower than for **SCNP**_**3**_. The ookinete population showed a strong shift to the region of higher fluorescent signals, originating from NPs in the case of **SCNP**_**3**_ as compared to the control, incubated only with the ookinete medium (Figure S7). The **SCNP**_**3**_ has a significantly higher ookinete binding as compared to all other SCNPs except for **SCNP**_**2**_. The results indicate that a charge-dependent targeting of ookinetes by SCNPs occurs and that there is an optimum surface charge because **SCNP**_**4**_ displays a lower binding rate.

**Figure 5:**
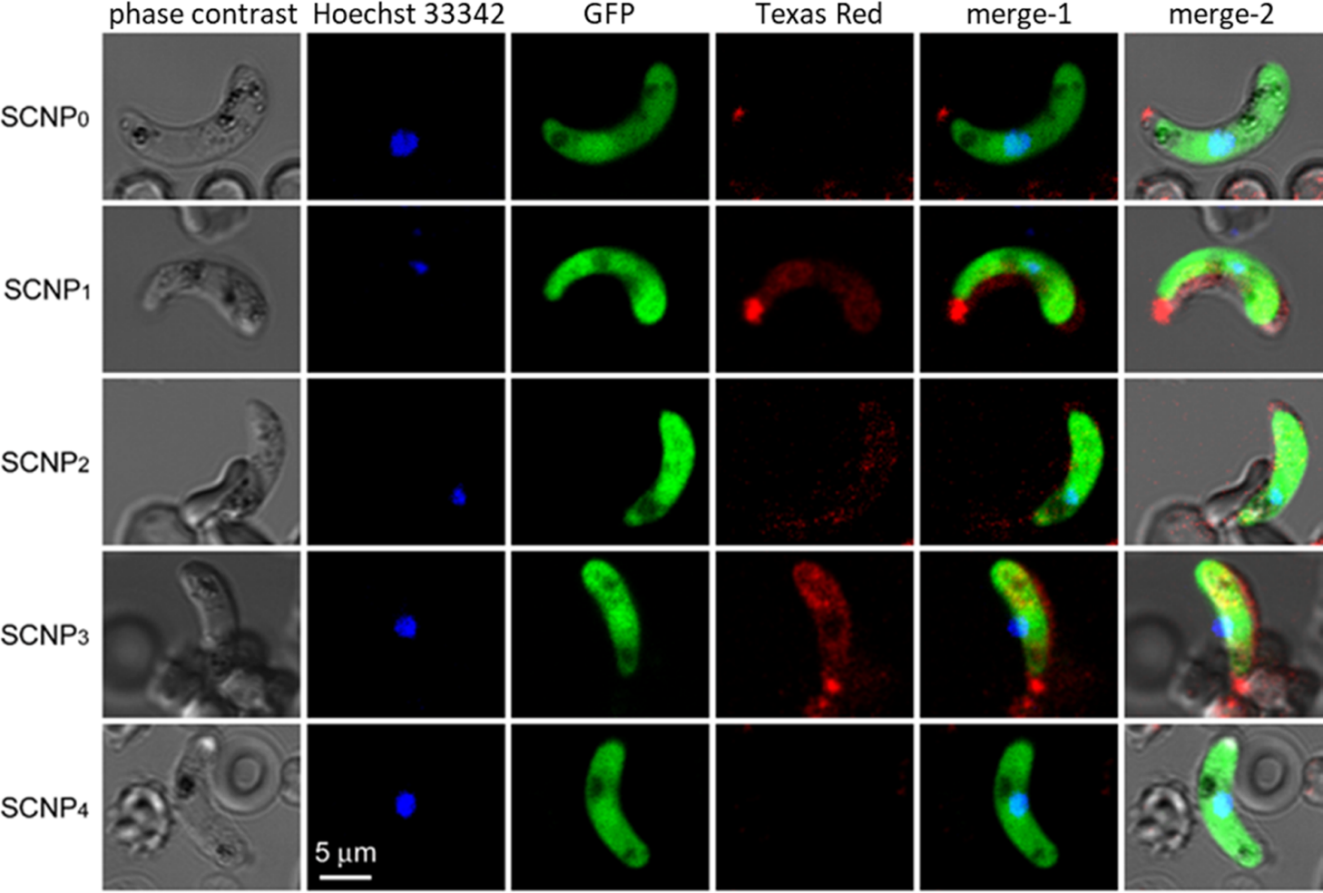
Representative confocal fluorescence images of SCNP binding to ex vivo-produced ookinetes. The *P. berghei* CTRP-GFP strain used in this assay expresses GFP only upon reaching the ookinete stage. Merge-1 combines the fluorescence channels only. Merge-2 combines the overlay of Merge-1 with the phase contrast image. The slight displacements observed in some cases between fluorescence and phase contrast images is due to cell displacement between different exposures, since cells were imaged alive to avoid fixation artifacts. Selected images correspond to individual confocal sections through the cell nucleus. Scale bar represents 5 μm.

The internalization of SCNPs in ookinetes was further evaluated by confocal fluorescence imaging. Ookinetes expressing GFP were counterstained with Hoechst, as shown in the control image Figure S8. In uptake control, Cy5-labeled heparin was used to validate targeting, as heparin has been shown earlier to bind ookinetes.^25^ The red signal corresponding to heparin is co-localized with the GFP signal from the ookinetes (Figure S9). **SCNP**_**0**_ shows no Texas Red signal in the ookinetes, the same is observed for **SCNP**_**4**_, these two samples had the lowest targeting shown by flow cytometry, and the weak interaction was not detected by CLSM (see Figure 5). In agreement with flow cytometry, the strongest ookinete uptake was observed for **SCNP**_**3**_.

**Figure 6:**
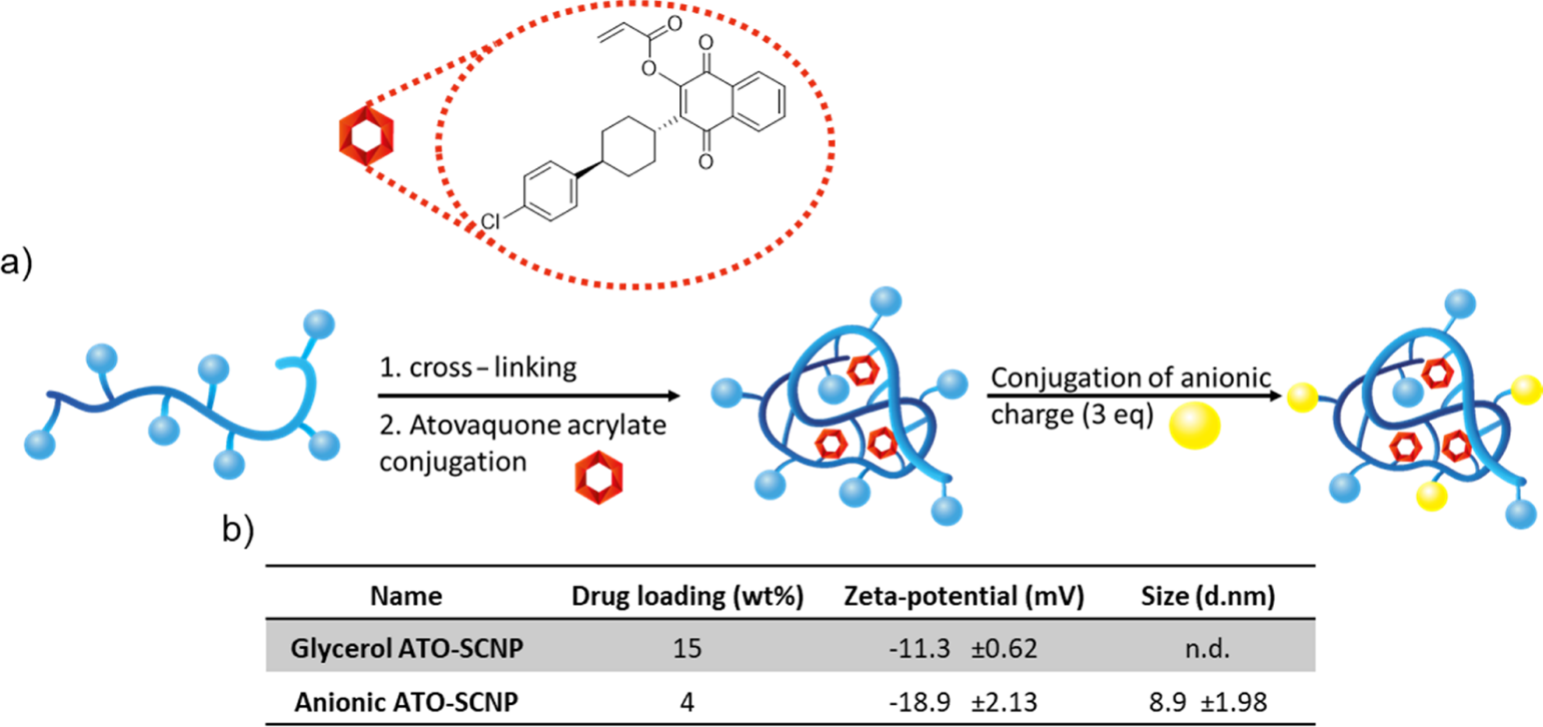
(a) Scheme of ATOA conjugation onto SCNPs and (b) table of ATO-SCNP characterization.

Having established the targeting capabilities of SCNPs against the ookinete state of the malaria parasite, we set out to employ SCNPs as a nanocarrier. Atovaquone, a powerful antimalarial agent, was, therefore, conjugated through a linker onto the SCNPs. El Hage et al. have studied various atovaquone derivates, observing that the derivate with an ester linkage on the alcohol moiety of atovaquone provides the highest activity against the growth of *Plasmodium falciparum* in vitro.^38^ An atovaquone prodrug was synthesized by the conjugation of acryloyl chloride to the alcohol moiety of atovaquone, the reaction was followed by ^1^H NMR spectroscopy (see Figure S10). In Figure 6a, the reaction steps to conjugate the prodrug onto the SCNPs and introduction of negative charges on the particles as described above are shown. During SCNP formation, free thiols on the polymers are intramolecularly cross-linked with bifunctional acrylates in a thiol-Michael addition and afterward residual-free thiols are typically end-capped by an acrylate (e.g., *N*,*N*-dimethylaminoethyl acrylate). For prodrug conjugation onto the SCNPs, precursor polymers with a higher xanthate content of 20% was employed. The xanthate content was partially used for cross-linking (10%), in accordance with the set of anionic SCNPs for targeting. Afterward, atovaquone acrylate (ATOA) was added in excess to the reaction end-capping all remaining free thiols forming ATO-SCNPs.

Successful conjugation of atovaquone onto the SCNPs was observed by ^1^H NMR spectroscopy, the signals observed in the aromatic region are associated with atovaquone, see Figure S11. Furthermore, the absence of signals around 6.2 to 6.7 ppm indicate the complete removal of the acrylate from the starting ATOA, confirming conjugation of atovaquone on the ATO-SCNPs. In the SEC trace, only a single signal is observed for the ATO-SCNP, see Figure S12. Furthermore, the SEC traces of the polymer and ATO-SCNPs showed a size reduction after the particle cross-linking. Indicating that the atovaquone loading did not significantly increase the SCNP size. Drug loading was determined by UV spectroscopy and resulted in 15 wt %, as shown in Figure 6b. For optimal ookinete targeting, the ATO-SCNPs were further functionalized with 3 equiv succinic anhydride. The final anionic ATO-SCNPs possess a lower drug loading of 4 wt %; however, this can mostly be ascribed to the overall molecular weight increase of the anionic ATO-SCNPs due to the succinate moieties. In Figure 6b, the zeta potentials of glycerol and anionic ATO-SCNPs are presented, showing a decrease in surface charge upon functionalization of the glycerol ATO-SCNPs with succinate groups, leading to a surface charge of −18.9 mV for anionic ATO-SCNP. Furthermore, the particle size of the anionic ATO-SCNP was measured by DLS revealing a diameter of 8.9 nm, which is in line with earlier measurements. Drug release from anionic ATO-SCNPs in water and under acidic conditions was studied over a course of 24 h incubation. The UV–vis spectra of the precipitate from anionic ATO-SCNPs shows an increase in the signal of the sample incubated in acidic conditions compared to the sample incubated in water (see Figure S13).

**Figure 7:**
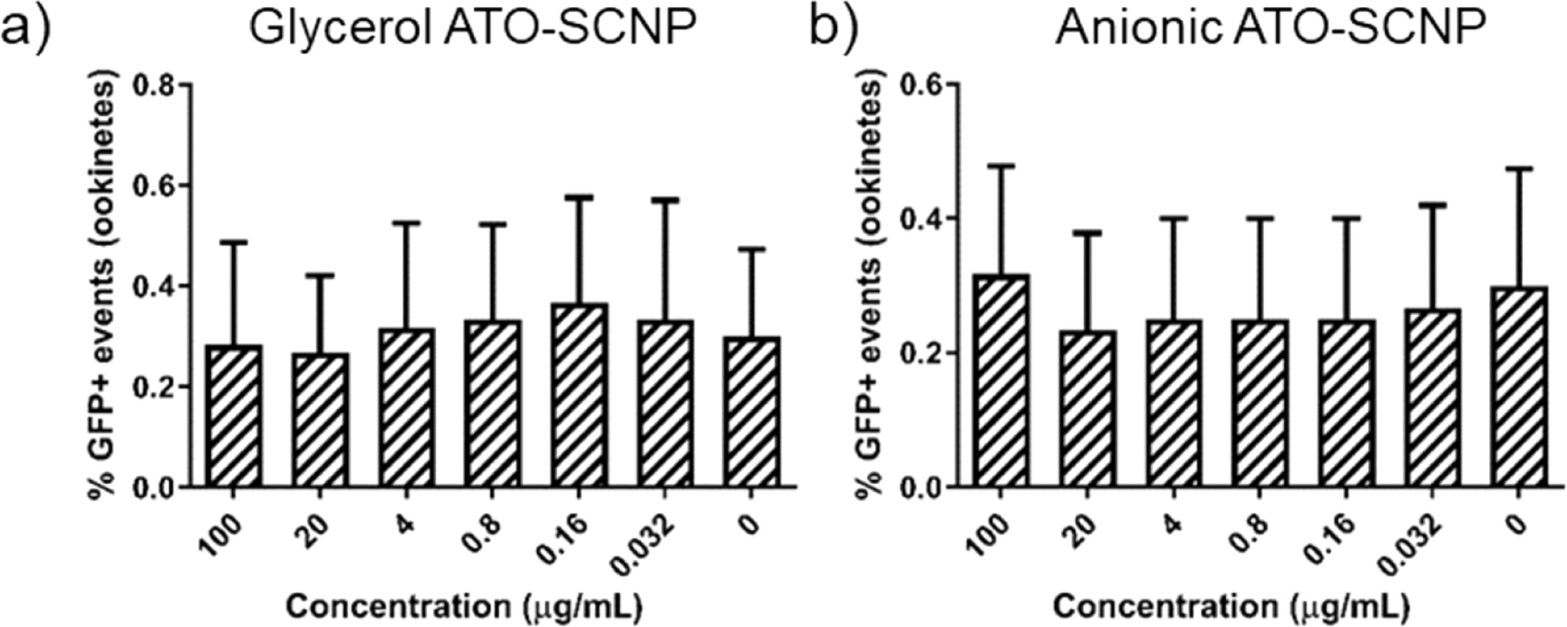
Ookinete population after incubation with (a) glycerol ATO-SCNP and (b) anionic ATO-SCNPs in the ookinete maturation assay using GFP expressing ookinetes measured by flow cytometry.

Drug delivery of atovaquone by SCNPs was evaluated in an ex vivo ookinete maturation inhibition assay. In Figure 7, the percentage of zygotes and ookinetes formed in the populations as measured by flow cytometry are presented after incubation with either ATO-SCNP or anionic ATO-SCNP. The results, however, show no significant decreases in ookinete maturation when atovaquone-loaded SCNPs are administered. This may have several causes. First, the anionic charge of the ATO-SCNPs is lower as compared to the SCNPs used in the targeting assays, which might result in decreased ookinete targeting. Higher drug loading might be required to give an observable decrease in ookinete maturation. Further, the linkage connecting atovaquone to the SCNPs might not readily degrade in the ookinetes.

## Conclusions

In conclusion, glycerol SCNPs were successfully synthesized and functionalized with succinic anhydride to introduce anionic surface charges. The resulting set of identically sized, but differently (negatively) charged SCNPs enabled systematic investigation of the influence of surface charge on ookinete targeting. Targeting assays revealed a pronounced effect of SCNP surface charge on their binding to ookinetes, with stronger anionic surface charges leading to increased targeting efficiency. The internalization of SCNPs with strong anionic surface charge was confirmed by confocal fluorescence microscopy. This together with the observed exclusive presence of SCNPs in mosquito midguts after feeding emphasizes the potential of SCNPs for targeting ookinete in mosquitos. This suggests that effective internalization of SCNPs in ookinetes can be achieved by generic targeting strategies such as negative charges. Successful conjugation of the anti-malarial atovaquone onto SCNPs through a prodrug approach was demonstrated. However, the resulting ATO-SCNPs did not exhibit significant inhibition of ookinete formation and therefore, alternative atovaquone conjugation for improved drug release will be evaluated in the future.

## Materials and Methods

### Materials

Hydrazine monohydrate (98%), 2-(dimethylaminoethyl) acrylate (DMAEA, 98%), PEGDA(*M*_n_ 258 g/mol), tris(2-carboxyethyl)phosphine hydrochloride (TCEP, ≥98%), succinic anhydride (>99.9%, Fluka), dimethyl sulfoxide (DMSO, anhydrous, 99.9%), acetic acid (≥99.8%), and pyridine (>99%) were purchased from Sigma-Aldrich. *N*,*N*-Dimethylformamide (DMF, ≥99.9%) was purchased from VWR. Triethyl amine (≥99%), 5-(4,6-dichlorotriazinyl) aminofluorescein (DTAF), and Texas Red C-dichlorotriazine (Texas Red) were purchased from Thermo Fisher Scientific. All chemicals were used without further purification unless stated otherwise. A co-polymer of solketal methacrylate (2,2-dimethyl-1,3-dioxolan-4-yl)methyl methacrylate, SMA) and 2-(ethyl xanthate) ethyl methacrylate (XMA) was prepared following a literature procedure.^32^ When stated as dry, solvents were treated with molecular sieves (4 Å) 24 h before use. SnakeSkin dialysis tubing (10k molecular weight cut-off) from Thermo Fisher Scientific was employed for dialysis.

DLS measurements were carried out in a 10 mM NaCl solution on Malvern Instruments Zetasizer ZS and the samples were filtered using GE Healthcare Whatman SPARTAN 13/0.2 RC 0.2 μm syringe filters prior to measurements. ^1^H NMR (400 MHz) spectra were recorded on a Bruker 400 spectrometer and chemical shifts were reported in ppm and referenced to DMSO. Gel permeation chromatography (GPC) was performed on a Waters e2695 Separations Module equipped with an Agilent PLgel 5 μm MIXED-D 300 × 7.5 mm column and Waters photodiode array detector (PDA 2998), fluorescence detector (FLR 2475), and RI detector (RI 2414). DMF (50 mM LiCl) was employed as an eluent and molecular weights (*M*_n_: number-average-molecular weight) were calibrated relative to PEO/PEG (DMF). GPC Samples were prepared in DMF followed by filtration using GE Healthcare Whatman SPARTAN 13/0.2 RC 0.2 μm syringe filters. Scanning transmission electron microscopy (STEM) images were recorded using a Zeiss Merlin HR-SEM with an add-on STEM detection system. Samples were prepared by adding 4 μL of the sample solution in double deionized water (MilliQ water purification system) on formvar coated copper grids and incubated for 30 s. The remaining solution was removed with a filter paper. As a staining solution, 4 μL of a 1% (w/v) uranyl acetate solution was incubated for 1 min.

### Animals

For assays involving the use of mice, in the presence of toxic effects, including, among others, >20% reduction in weight, aggressive, and unexpected animal behavior or the presence of blood in feces, animals were immediately anesthetized using a 100 mg/kg Ketolar plus 5 mg/kg Midazolan mixture and sacrificed by cervical dislocation. The animal care and use protocols followed adhered to the specific national and international guidelines in accordance with the current Catalan (D 214/1997/GC) and Spanish laws (RD 53/2013; order ECC/566/2015) and the corresponding European Directive (2010/63 EU).

### Glycerol SCNP Formation

Glycerol SCNPs were prepared as previously described (see Figure S1).^32^ In brief, a co-polymer (**P1**) [p(XMA–SMA), 500 mg] of glycerol and xanthate methacrylate groups (0.35 mmol eq thiol monomer) was deprotected using hydrazine (34.4 μL, 0.7 mmol, 2 equiv) in 10 mL DMSO, yielding free thiols. After filtration, the co-polymer was added dropwise to a dilute solution of PEGDA (258 g/mol, 86.8 μL, 0.35 mmol, 1 equiv) and TCEP (18.5 mg, 0.06 mmol, 0.2 equiv) to facilitate the intramolecular cross-linking by thiol-Michael addition. Remaining thiols were end-capped with 1.9 mL DMAEA (12.4 mmol) and the particles were dialyzed. The final SCNPs were obtained by lyophilization as a white powder (∼300 mg).

### Negatively Charged SCNPs

The functionalization of glycerol-SCNPs with a negative charge was achieved as previously described^39^ and a range of negative SCNPs was prepared by increasing the equivalents of succinic anhydride (see Figure S2). As an example for **SCNP**_**1**_, glycerol-SCNPs (40 mg, 0.2 mmol in glycerol units) were dissolved in 7 mL DMSO, succinic anhydride (20.8 mg, 0.2 mmol, 1 equiv), and pyridine (16.8 μL, 0.2 mmol, 1 equiv) were added to the solution. The reaction was stirred at 100 °C overnight and subsequently dialyzed. The product was obtained by lyophilization (∼20 mg).

### Fluorescent Labeling of SCNPs

The SCNPs were labeled with either DTAF or Texas Red using the same procedure, here described for Texas Red. To a solution of **SCNP**_**0**_ to **SCNP**_**5**_ (20 mg, 0.1 mmol in glycerol units) in 5 mL carbonate-bicarbonate buffer (CB buffer, 0.1 M sodium carbonate/sodium bicarbonate, pH 9–10), 1.66 mg of Texas Red C-dichlorotriazine (Texas Red, 0.002 mmol, 0.02 equiv) was added and stirred overnight. Subsequently, the product was purified first by dialyzing for 3 days. Remaining free dye was removed using a PD-10 column. The particles were obtained by lyophilization (∼10 mg).

### Atovaquone Acrylate

To a stirred solution of atovaquone (200 mg, 0.55 mmol) in dichloromethane (8 mL) under a nitrogen atmosphere, triethylamine (92 μL, 0.66 mmol) was added and the solution was cooled on an ice bath. Acryloyl chloride (53 μL, 0.66 mmol) was added dropwise and the solution was stirred overnight while warming up to room temperature. The mixture was diluted with dichloromethane (15 mL) and washed with water (2 × 5 mL) and brine (1 × 5 mL), dried over MgSO_4_, and concentrated under reduced pressure. The residue was purified by flash column chromatography (silica gel, heptane/dichloromethane 2:1 to give ATOA as yellow crystals in 140 mg yield (60%).

^1^H NMR (400 MHz, CDCl_3_): δ (ppm) 8.08, 7.75, 7.28 and 7.16 (m, 8H, ArH), 6.74, (d, 1H, CH_2_=CH) 6.45 (m, 1H, CH=CH_2_), 6.17 (d, 1H, CH_2_=CH), 3.11 (m, 1H, (CH_2_)_2_–CH–PhCl), 2.58 (m, 1H, (CH_2_)_2_–CH–C=C), 1.98 (m, 4H, CH_2_–CH_2_–CH–PhCl), 1.85 (m, 2H, CH_2_–CH_2_–CH–C=C) and 1.55 (m, 2H, CH_2_–CH_2_–CH–C=C).

### Atovaquone-Loaded ATO-SCNPs

**P1** (100 mg, 0.11 mmol eq thiol monomer) was dissolved in DMSO (2 mL) and purged with nitrogen. Hydrazine (11.1 μL, 0.23 mmol, 2 equiv) was added and the solution was stirred for 30 min. A solution of 50 mL CB buffer with TCEP (5.96 mg, 0.02 mmol, 0.2 equiv) and PEGDA (13.9 μL, 0.06 mmol, 0.5 equiv) was prepared in a three-necked round-bottomed flask and purged with N_2_. The filtered polymer solution was added through a dropping funnel to a buffer solution under continuous stirring. After 4 h of stirring, ATOA (46.5 mg, 0.11 mmol, 1 equiv) dissolved in DMSO (3 mL) was added to the solution and stirred overnight. The product was subsequently dialyzed against water and lyophilized (∼60 mg).

### Atovaquone-Loaded Anionically Charged SCNPs

Atovaquone-loaded SCNPs (40 mg, 0.18 mmol glycerol units) were dissolved in DMSO (7 mL), succinic anhydride (54.9 mg, 0.55 mmol, 3 equiv), and pyridine (43.4 mg, 0.55 mmol, 3 equiv) were added to the solution. The reaction mixture was stirred overnight under reflux. The product was subsequently dialyzed against water for 3 days and lyophilized (∼20 mg).

### Atovaquone Release from Anionic ATO-SCNPs

Anionic ATO-SCNPs were incubated at 5 mg/mL in 1 M acetic acid or demi water and stirred for 24 h. Drug release was measured by UV–vis taking samples of the precipitated ATO in DMSO.

### Biodistribution Study of SCNP_0_ in Mosquitos

Three feeding cups, one control and two experiments, were prepared each containing 6 female *Anopheles gambiae* mosquitos. Mosquitos were allowed to feed for 1 day from 10% sucrose by a syringe feeder. On day 2, the 10% sucrose solution was replaced in the two experiment cups with each NP solution at 0.25 mg/mL in 10% sucrose. In the control cup, the mosquitos continued to feed with 10% sucrose. After 3 days of feeding with NPs, the mosquitos were dissected and fixed in 4% formaldehyde in 1× phosphate buffered saline (PBS). Samples were observed in a fluorescence stereoscope.

### Ex Vivo Production of Ookinetes and SCNP Targeting

To evaluate the ookinete targeting of Texas Red-labeled SCNPs, ookinetes were produced ex vivo following a protocol previously reported.^25,37^ Briefly, *P. berghei* CTRP-GFP^37^ parasites were administered intraperitoneally (i.p.) to a BALB/c mouse (Janvier Laboratories, Le Genest-Saint-Isle, France). Four days later, this animal was used as a donor to infect i.p. a second mouse (M2) that was previously treated with phenylhydrazine (120 μL of a 10 mg/mL solution in PBS) to enhance reticulocyte production. Once infection was established in M2, blood carrying gametocytes was collected by intracardiac puncture and immediately diluted in 30 mL of ookinete medium [10.4 g/L Roswell Park Memorial Institute medium (RPMI) supplemented with 2% w/v NaHCO_3_, 0.05% w/v hypoxantine, 0.02% w/v xanthurenic acid, 50 U/mL penicillin, 50 μg/mL streptomycin, 20% heat-inactivated fetal bovine serum (Invitrogen, US), and 25 mM HEPES, pH 7.4]. Finally, the culture was incubated under orbital shaking (50 rpm) for 24 h at 21 °C to allow ookinete conversion. After this time, 0.5 mL of ookinete culture were incubated with 0.5 mg/mL of each SCNPs in an ookinete medium for 1 h at room temperature. Then, samples were washed three times with PBS and nuclei were counterstained with Hoechst 33342 (2 μg/mL). As a control, Cy5-labeled heparin (Nanocs Inc., New York, US; λ_ex_/λ_em_: 650/670 nm) was included to validate targeting. Images were acquired with a Zeiss LSM880 confocal fluorescence microscope (Jena, Germany). Hoechst 33342 was excited with a 405 nm diode laser and Texas Red with a 633 nm line of a helium-neon laser. To avoid crosstalk between the different fluorescence signals, a sequential scanning was performed. Each experiment was repeated on at least three biological replicates.

For flow cytometry analysis, ookinetes incubated with SCNPs were washed and diluted 1:10 in PBS. Afterward, samples were analyzed in a LSRFortessa flow cytometer (BD Biosciences, San Jose, CA, US) set up with the 5 lasers and 20 parameters standard configuration. GFP and Texas Red fluorochromes were excited using 488 and 561 nm lasers, and their respective emissions collected with 525/40 and 610/20 nm filters. The GFP-positive population was selected and analyzed by its Texas Red intensity using Flowing Software 2.5.1 (www.btk.fi/cell-imaging; Cell Imaging Core, Turku Centre for Biotechnology, Finland). GraphPad Prism 8 (GraphPad Software, San Diego, USA) was used to plot the histograms. Statistical analysis of the flow cytometry data was performed with SPSS 22, using one-way analysis of variance (ANOVA) with Tukey post-hock analysis. Classifications of the differences were described as the following: significant (*p* < 0.05), very significant (*p* < 0.01), and extremely significant (*p* < 0.001).

### Ookinete Maturation Assay in Presence of Atovaquone-Loaded SCNPs

To evaluate the effect of atovaquone-loaded SCNPs, an ookinete maturation inhibition assay was carried out. Briefly, blood extracted from three infected mice was used to establish cultures of *P. berghei* CTRP-GFP (which expresses GFP in the ookinete stage). The resulting gametocyte-containing culture was plated in 96-well plates and treated with increasing concentrations of SCNPs containing 15 or 4 wt % atovaquone. To determine the tested concentrations, the IC_50_ of atovaquone for *P. falciparum* blood stages (0.036 μg/mL) was taken as reference.^40^ Hence, 0.032, 0.16, 0.8, 4, 20, and 100 μg/mL were tested for each sample in duplicates. Plates were incubated for 24 h at 21 °C under continuous stirring to allow ookinete production ex vivo, and the % of GFP positive events in each well was analyzed by flow cytometry as described above.

## Supporting Information Available

DLS data, ^1^H NMR spectra, SEC data, flow cytometry data and confocal microscopy images, and UV–vis data regarding drug conjugation and release (PDF)
