# Supplementary material for: Single-Chain Polymer Nanoparticles Targeting the Ookinete Stage of Malaria Parasites
Source: ACS Infect Dis. 2022 Dec 14;9(1):56–64. doi: 10.1021/acsinfecdis.2c00336 (PMC9841520; doi:10.1021/acsinfecdis.2c00336)
Supplement: Supplementary file 1 — id2c00336_si_001.pdf [file id2c00336_si_001.pdf]

Supporting Information

Single-Chain Polymer Nanoparticles Targeting the Ookinete Stage of Malaria Parasites

Naomi M. Hamelmann,[a] Jan-Willem D. Paats,[a] Yunuen Avalos-Padilla,[b,c] Elena Lantero,[b,c] Lefteris Spanos,[d] Inga Siden-Kiamos,[d] Xavier Fernàndez-Busquets[b,c,e] and Jos M. J. Paulusse[a]

[a] Department of Molecules and Materials, MESA+ Institute for Nanotechnology and TechMed Institute for Health and Biomedical Technologies, Faculty of Science and Technology, University of Twente, P.O. Box 217, 7500 AE Enschede, The Netherlands.

[b] Institute for Bioengineering of Catalonia (IBEC), The Barcelona Institute of Science and Technology, Baldiri Reixac 10–12, ES-08028 Barcelona, Spain

[c] Barcelona Institute for Global Health (ISGlobal, Hospital Clínic-Universitat de Barcelona), Rosselló 149-153, ES-08036 Barcelona, Spain

[d] Institute of Molecular Biology and Biotechnology, FORTH, N. Plastira 100, 700 13 Heraklion, Greece

[e] Nanoscience and Nanotechnology Institute (IN2UB, Universitat de Barcelona), Martí i Franquès 1, ES-08028 Barcelona, Spain

E-mail: inga@imbb.forth.gr; xfernandez@ibecbarcelona.eu; j.m.j.paulusse@utwente.nl

Table of contents

Figures of SCNP synthesis and functionalization S-3

DLS data S-3

STEM image S-4

^{1}H NMR spectra S-4

GPC traces S-5

FACS populations S-5

CLSM image S-5

CLSM images S-6

^{1}H NMR spectra S-6

GPC traces S-7

UV-Vis spectra S-7

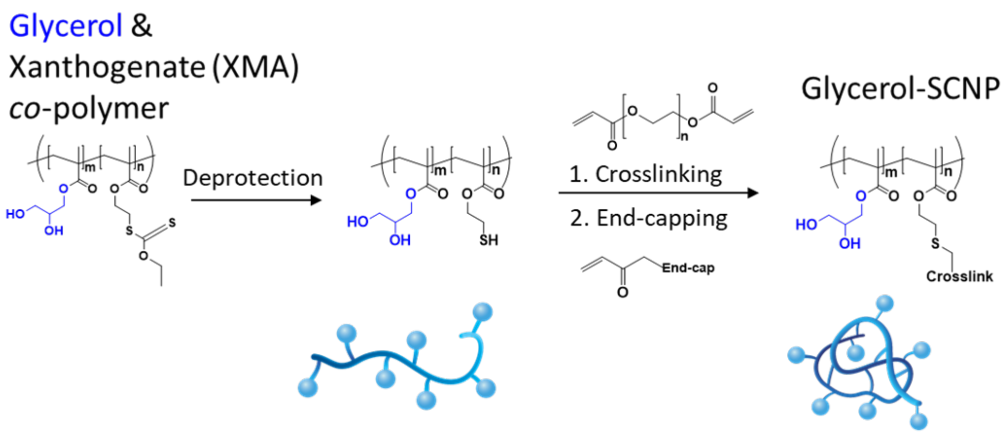

Figure S1. Synthesis of glycerol SCNPs with schematic presentation of the SCNPs.

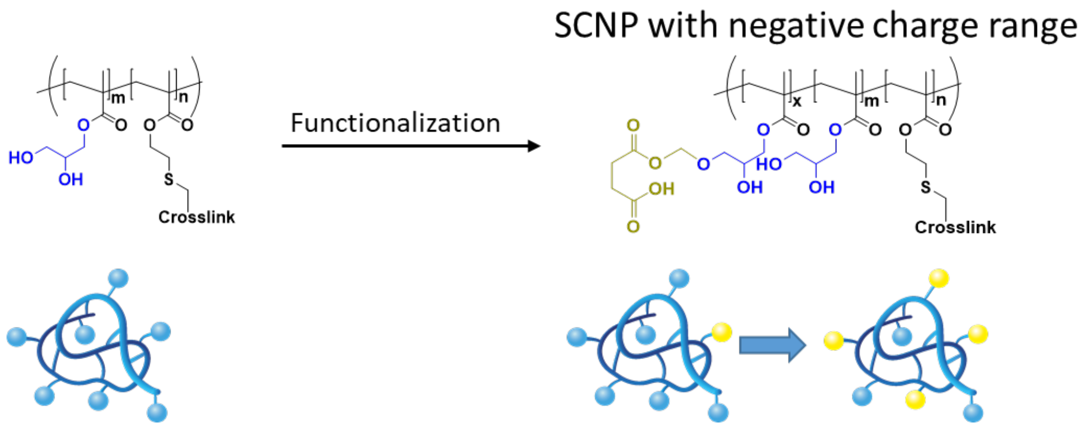

Figure S2. Functionalization glycerol SCNPs with succinic anhydride with the resulting charge range.

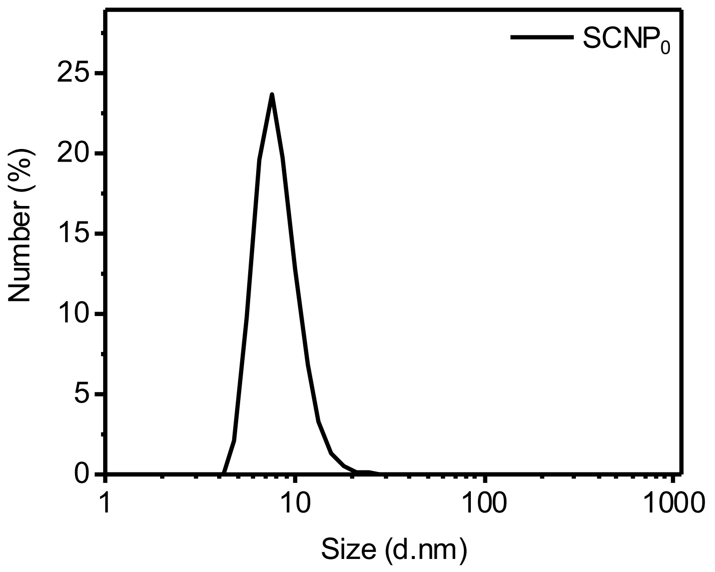

Figure S3. DLS analysis of **SCNP$_0$**.

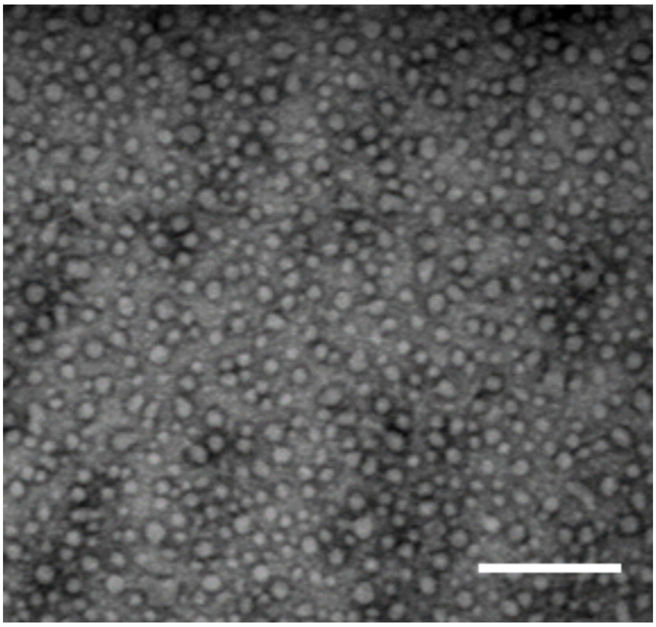

Figure S4. STEM image of **SCNP$_0$** (scale bar is 200 nm).

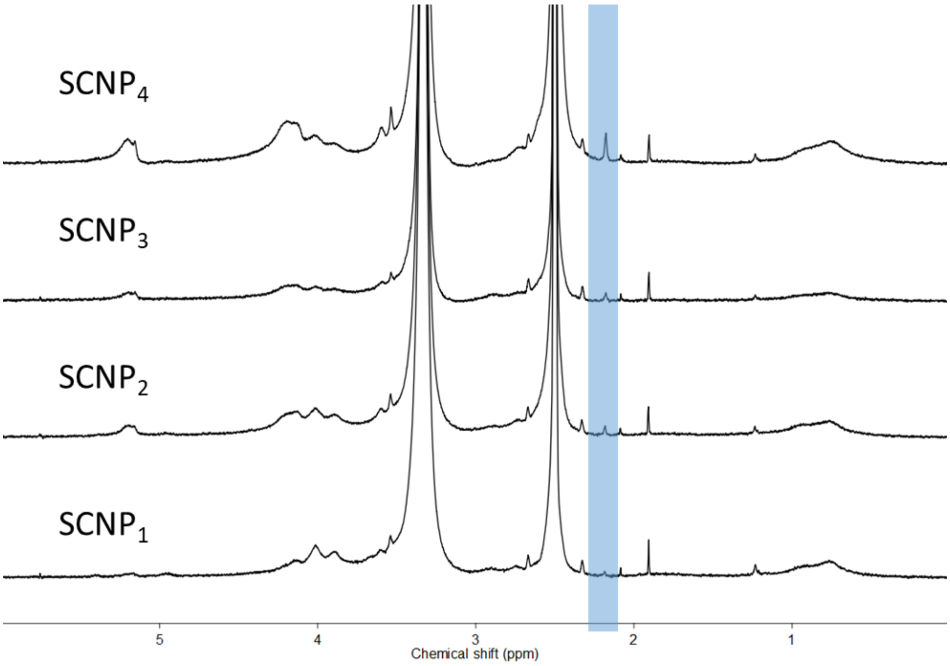

Figure S5. Overlay of ^{1}H NMR spectra of functionalized **SCNP$_1$** to **SCNP$_4$** in DMSO-d$_6$. Blue bar indicates increasing signal upon increased functionalization with succinic anhydride.

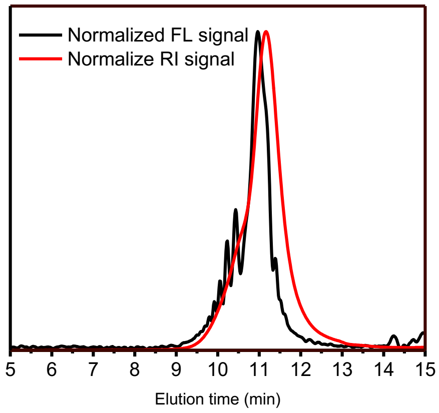

Figure S6. Overlay of GPC traces of $\mathbf{SCNP_0}$ conjugated with Texas red (em. 598 nm).

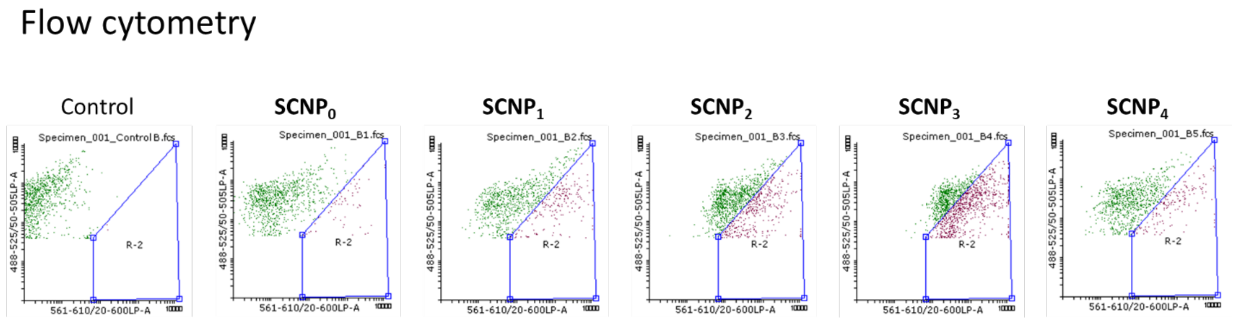

Figure S7. Sample populations of ex vivo ookinete targeting assay measured by FACS.

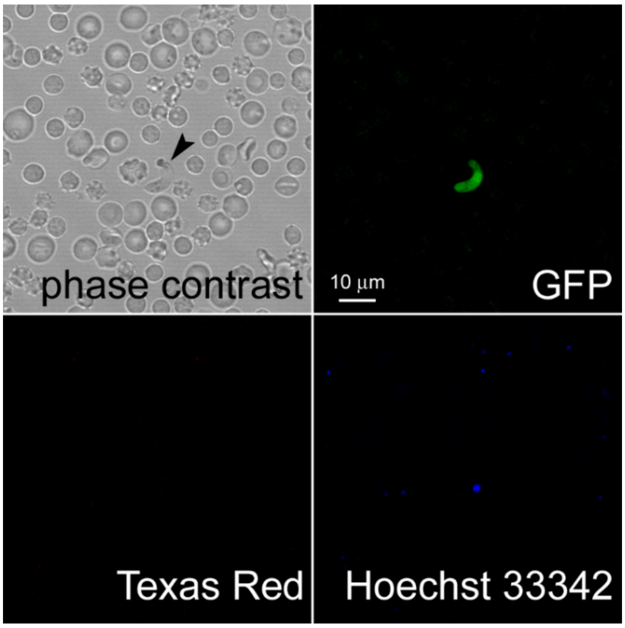

Figure S8. Representative confocal fluorescence microscopy image of an ex vivo-produced ookinete in the absence of a red fluorochrome. The arrowhead indicates a *Plasmodium berghei* ookinete from the CTRP-GFP strain used in this assay (which expresses GFP only when reaching the ookinete stage) among erythrocytes and other blood components, to show the absence of unspecific fluorescence in the red channel. The microscope and camera settings are identical to Figure 5. Scale bar represents 10 µm.

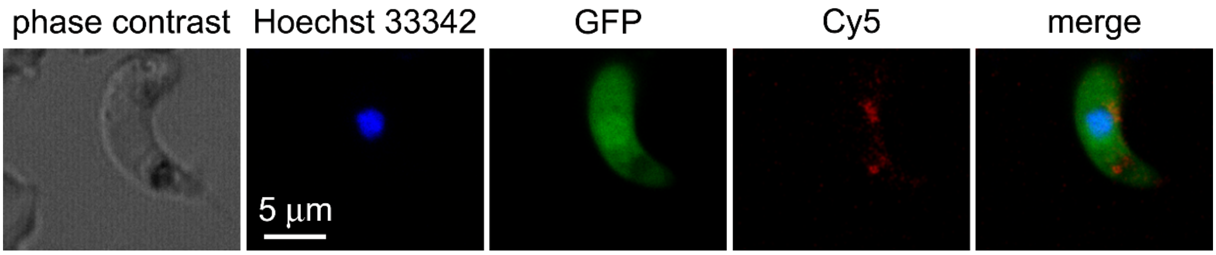

Figure S9. Representative confocal fluorescence microscopy image of Cy5-labeled heparin binding to an ex vivo-produced ookinete. The *Plasmodium berghei* CTRP-GFP strain used in this assay expresses GFP only when reaching the ookinete stage. Scale bar represents 5 µm.

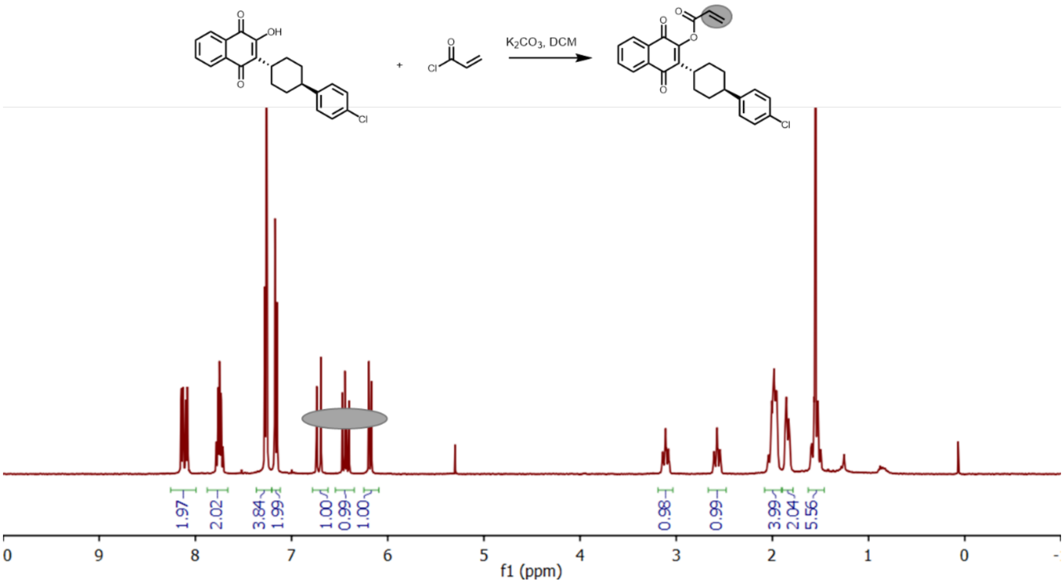

Figure S10. ^{1}H NMR spectra of the prodrug atovaquone acrylate (ATOA).

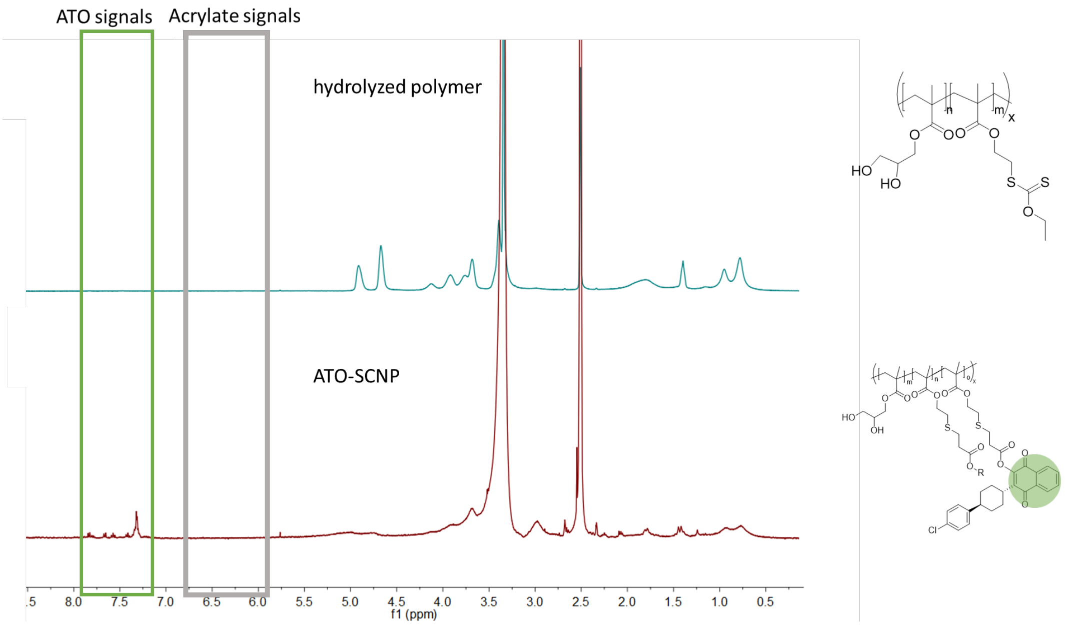

Figure S11. ^{1}H NMR of the co-polymer (top) and the atovaquone conjugated SCNP (bottom) in d6-DMSO.

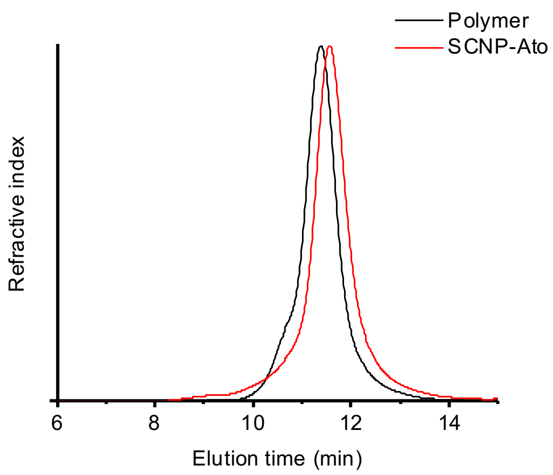

Figure S12. Size exclusion chromatography graph of the precursor polymer and the ATO-SCNPs, measured in DMF.

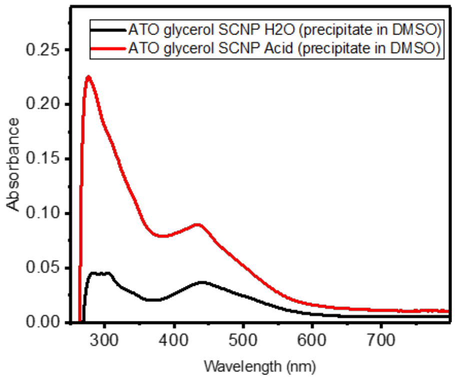

Figure S13. UV-Vis spectra of ATO release measured from the precipitate of anionic ATO-SCNPs incubated in either demi water or 1 M acetic acid for 24 h in DMSO.
